# Formulaic Language Resources May Help Overcome Difficulties in Speech-Motor Planning after Stroke

**DOI:** 10.1371/journal.pone.0233608

**Published:** 2020-06-04

**Authors:** Benjamin Stahl, Bianca Gawron, Frank Regenbrecht, Agnes Flöel, Sonja A. Kotz

**Affiliations:** 1 Department of Neurology, University Medicine Greifswald, Greifswald, Germany; 2 Department of Neurology, Charité Universitätsmedizin Berlin, Berlin, Germany; 3 Max Planck Institute for Human Cognitive and Brain Sciences, Leipzig, Germany; 4 Psychologische Hochschule Berlin, Berlin, Germany; 5 Department of Speech Science, Martin-Luther-Universität Halle-Wittenberg, Halle, Germany; 6 Clinic for Cognitive Neurology, University Hospital Leipzig, Leipzig, Germany; 7 German Center for Neurodegenerative Diseases, Rostock and Greifswald, Germany; 8 Department of Neuropsychology and Psychopharmacology, Faculty of Psychology and Neuroscience, Maastricht University, Maastricht, The Netherlands; San Diego State University, UNITED STATES

## Abstract

**Purpose:**

Decades of research have explored communication in cerebrovascular diseases by focusing on formulaic expressions (e.g., “Thank you”—“You’re welcome”). This category of utterances is known for engaging primarily right-hemisphere frontotemporal and bilateral subcortical neural networks, explaining why left-hemisphere stroke patients with speech-motor planning disorders often produce formulaic expressions comparatively well. The present proof-of-concept study aims to confirm that using verbal cues derived from formulaic expressions can alleviate word-onset difficulties, one major symptom in apraxia of speech.

**Methods:**

In a cross-sectional repeated-measures design, 20 individuals with chronic post-stroke apraxia of speech were asked to produce (i) verbal cues (e.g., /guː/) and (ii) subsequent German target words (e.g., “Tanz”) with critical onsets (e.g., /t/). Cues differed, most notably, in aspects of formulaicity (e.g., stereotyped prompt: /guː/, based on formulaic phrase “Guten Morgen”; unstereotyped prompt: /muː/, based on non-formulaic control word “Mutig”). Apart from systematic variation in stereotypy and communicative-pragmatic embeddedness possibly associated with holistic language processing, cues were matched for consonant-vowel structure, syllable-transition frequency, noun-verb classification, meter, and articulatory tempo.

**Results:**

Statistical analyses revealed significant increases in correctly produced word onsets after verbal cues with distinct features of formulaicity (e.g., stereotyped *versus* unstereotyped prompts: *p* < 0.001), as reflected in large effect sizes (Cohen’s *d*_*z*_ ≤ 2.2).

**Conclusions:**

The current results indicate that using preserved formulaic language skills can relieve word-onset difficulties in apraxia of speech. This finding is consistent with a dynamic interplay of left perilesional and right intact language networks in post-stroke rehabilitation and may inspire new treatment strategies for individuals with apraxia of speech.

## Introduction

Left-hemisphere stroke patients often experience a profound deficit in speech-motor planning, a syndrome especially apparent in the production of word onsets (e.g., incorrect phoneme /k/ in “Coat”) [1]. Known as apraxia of speech, this syndrome frequently occurs alongside other neurological communication disorders, including aphasia. In everyday life, apraxia of speech constrains the ability to interact verbally, and therefore, probably adds to the risk of depressive episodes in stroke patients with communication disorders [2].

Given the repeated failure in the production of critical word onsets (e.g., incorrect /k/ in the *literal* use of “Cool”), it is striking how effortlessly some individuals with apraxia of speech perform the same phonemes as part of formulaic expressions (e.g., correct /k/ in the *conversational* use of “Cool”). By definition, such expressions differ from newly created, grammatical utterances in that they are (i) stereotyped in form and (ii) closely related to communicative-pragmatic context [3]. According to neurolinguistic research, formulaic expressions may be stored and retrieved in a holistic manner [4–6]. Although the proportion of formulaic expressions to spoken language varies with type of measure and discourse, these utterances are widely regarded as crucial to the success of social interaction in many communicative aspects of daily life [7]. While left perisylvian areas of the brain seem to support primarily propositional-grammatical utterances [8–10], processing of formulaic expressions proved to engage, in particular, right-hemisphere frontotemporal and bilateral subcortical neural networks [11–15]. This may account for anecdotal evidence implying that symptoms in apraxia of speech are more common in propositional-grammatical utterances and, in contrast, less pronounced in formulaic expressions. So far, surprisingly few attempts have been made to exploit intact speech-motor sequences within formulaic expressions to compensate for word-onset difficulties—an issue addressed in the present study.

To facilitate the production of phonemes, speech-language therapists typically provide a variety of cues by prompting word onsets in different modalities: audiovisually (i.e., speaking aloud and allowing lip-reading), tactile-kinesthetically (i.e., stimulating the patients’ articulatory organs) or gesturally (i.e., giving hand signs) [16]; further valuable approaches are available [17]. Complementing this repertoire of treatment methods, we propose an alternative strategy focusing on formulaic language resources in a two-step procedure. In step one, the therapists identify critical word onsets (e.g., incorrect /k/ in “Coat”) and formulaic expressions with intact corresponding phonemes (e.g., correct /k/ in “You’re welcome”). In step two, patients combine these phonemes (e.g., /jʊə ˈwɛl/-/kəʊt/) to prepare critical word onsets (e.g., /k/) in a repeated fashion until no more help is required (e.g., correct /k/ in “Coat”). Anecdotal evidence from pilot patients indicates that the suggested keyword technique can have an immediate positive impact on word-onset difficulties in moderate-to-severe apraxia of speech. However, these casual reports need to be substantiated by data demonstrating that the technique actually taps into language formulaicity. In fact, the postulated benefit from formulaic language resources may well result from other linguistic parameters, among them consonant-vowel structure, syllable-transition frequency, noun-verb classification, meter, and articulatory tempo.

Controlling for the above linguistic parameters, the current proof-of-concept study seeks to determine the effect of preserved formulaic language skills on word-onset difficulties in apraxia of speech. In a cross-sectional repeated-measures design, 20 left-hemisphere stroke patients with chronic moderate-to-severe apraxia of speech produced non-formulaic German target words after systematic presentation of verbal cues. These cues differed in distinct features of language formulaicity, as detailed below. Two phoneticians assessed the onsets of non-formulaic target words with regard to articulatory quality. Based on anecdotal evidence from pilot patients, we predict significant gains in articulatory quality if target words are preceded by cues derived from formulaic expressions.

## Materials and methods

### Participants

Recruitment was administered in collaboration with several Berlin rehabilitation centers and support groups for individuals with aphasia in the years between 2013 and 2018. After routine referral to the study team, we contacted the potential participants and invited them to a screening session to check their eligibility. Inclusion criteria were: moderate-to-severe apraxia of speech (main characteristics: phonetic distortions and phonemic errors; dysfluent speech with initiation problems, syllable segregation, inter-syllabic pausing, phoneme lengthening or continuous repairs; and effortful speech with obvious groping or struggling behavior), as diagnosed by two independent clinical linguists in analogy to previous work [18]; chronic stage of symptoms at least six months post-onset of stroke to minimize effects of spontaneous recovery over time; native speaker of German; intact right hemisphere to ensure relatively preserved formulaic language skills; and right-handedness according to the Edinburgh Handedness Inventory [19]. Exclusion criteria were: apraxia of speech due to traumatic brain injury or neurodegenerative disease; dysarthria; and severe hearing disorder that may discourage patients from engaging in the testing sessions.

A total of 20 individuals agreed to participate in the present study, a patient sample determined in an *a-priori* power analysis (assumed effect size in a paired-sample *t*-test: Cohen’s *d*_*z*_ = 0.8; two-tailed significance level of *α* = 0.05; 1–*β* = 0.90; resulting *n* = 19; estimated drop-out rate: 5%; final *n* = 20) [20]. On average, patients were aged 59.8 years (standard deviation: 12.9 years) and 32.2 months post-onset of stroke (standard deviation: 24.5 months). Each patient had suffered a cerebrovascular accident with subsequent lesions in parts of the left frontal, parietal, and temporal lobes, as well as in adjacent subcortical areas. Aside from apraxia of speech—the prevailing disorder in our sample—all patients were diagnosed with mild-to-moderate aphasia, as confirmed by clinical records and the Aachen Aphasia Test [21]. The study was approved by the ethics review board at the Charité Universitätsmedizin Berlin, Germany, with written informed consent obtained from each patient (reference number: EA1/158/13; for individual case histories, see Table 1).

**Table 1:** Patient histories.

Patient	Gender	Age (in years)	Months after onset of stroke	Origin	Clinical diagnosis
01	Female	40	10	Left MCA ischemia	Severe AoS
02	Male	72	6	Left MCA ischemia	Severe AoS
03	Male	71	23	Left MCA ischemia	Moderate AoS
04	Male	49	6	Left MCA ischemia	Severe AoS
05	Male	45	23	Left MCA ischemia	Moderate AoS
06	Male	64	32	Left MCA ischemia	Moderate-to-severe AoS
07	Male	76	76	Left MCA ischemia	Severe AoS
08	Male	62	10	Left MCA ischemia	Moderate AoS
09	Male	70	30	Left MCA ischemia	Moderate AoS
10	Male	64	41	Left MCA ischemia	Severe AoS
11	Male	74	32	Left MCA ischemia	Severe AoS
12	Male	51	43	Left MCA ischemia	Severe AoS
13	Female	36	27	Left MCA ischemia	Moderate-to-severe AoS
14	Male	60	12	Left MCA ischemia	Severe AoS
15	Female	41	95	Left MCA ischemia	Moderate-to-severe AoS
16	Female	58	12	Left MCA ischemia	Severe AoS
17	Male	60	36	Left MCA ischemia	Severe AoS
18	Male	51	81	Left MCA ischemia	Moderate-to-severe AoS
19	Male	67	18	Left MCA ischemia	Moderate AoS
20	Female	84	31	Left MCA ischemia	Severe AoS
**Mean (SD)**		**59.8 (12.9)**	**32.2 (24.5)**		

MCA: Middle cerebral artery; AoS: Apraxia of speech; SD: Standard deviation

### Linguistic materials

As *target words*, we chose 10 non-formulaic one-syllable nouns from the middle-frequency spectrum (e.g., “Tanz” [/tants/; German for “dance”]). The critical onsets of the target words differed in manner and place of articulation to cover a large variability of errors in speech-motor planning (plosives: /d/, /t/, /g/, /k/; fricatives: /v/, /f/, /z/, /ʃ/, /ʁ/; nasal: /n/). To ensure comparability of target words, the critical onsets were always followed by the vowel /a/.

To identify suitable *formulaic expressions*, eight linguists contributed and assessed over 100 conversational phrases with regard to (i) stereotypy and (ii) embeddedness in communicative-pragmatic context [3]. Phrases were considered as formulaic only if they met these two criteria according to the linguists’ judgement. In addition, all phrases needed to be part of a formulaic repertoire generally known to native speakers [22]. Based on audiotaped sessions of pilot patients, two phoneticians rated the articulatory quality of critical syllable onsets within each formulaic expression (e.g., /t/ in “Guten Morgen” [/ˈɡuːtn̩ ˈmɔʁɡn̩/; German for “Good Morning”]; /g/ in “Entschuldigung” [/ɛntˈʃʊldɪɡʊŋ/; German for “I’m sorry”]; /z/ in “Wiedersehen” [/ˈviːdɐˌzeːən/; German for “Good bye”]; most phrases were “symmetrical” in the sense that they can be both salutation and reply). This procedure resulted in 16 formulaic expressions with widely preserved critical syllable onsets (e.g., intact /t/ in “Guten Morgen”), thus making it possible to use the previous segments as prompts (e.g., /guː/) to facilitate the production of specific phonemes (e.g., /t/).

Finally, we selected 16 *non-formulaic control words* (e.g., /muː/ from “Mutig” [/ˈmuːtɪç/; German for “brave”]) matched for linguistic parameters of the prompts originating from formulaic expressions (e.g., /guː/ from “Guten Morgen”). As suggested by the literature, relevant linguistic parameters were: number of syllables preceding a critical phoneme [23], syllable-transition frequency [24], consonant-vowel structure [25], noun-verb classification [26], and meter [27]. To prevent articulatory priming through consonant repetitions, none of the 32 prompts included the critical phoneme (e.g., not allowed: /guː/ preceding /g/). Moreover, none of the prompts prepared the critical phoneme through co-articulation (e.g., not allowed: /daŋ/ from “Danke” [/ˈdaŋkə/; German for “Thank you”] preceding /k/; for statistics of the linguistic materials, see Table 2).

**Table 2:** Linguistic materials.

	Number of syllables preceding the critical phoneme	Syllable frequency	Syllable-transition frequency
**Stereotyped prompts (SD)**	1.6 (0.6)	3167 (7807)	153 (174)
**Unstereotyped prompts (SD)**	1.6 (0.6)	6310 (16907)	173 (200)

Means of linguistic parameters for stereotyped (e.g., /guː/) and unstereotyped prompts (e.g., /muː/) serving as verbal cues to facilitate the production of critical word onsets (e.g., /t/ in “Tanz”). Frequency characteristics are extracted from the CELEX database, with values referring to the total number of occurrence in German [28]. Syllable frequency reflects prompts (e.g., /guː/ or /muː/) without considering subsequent critical phonemes (e.g., /t/). Syllable-*transition* frequency represents prompts *and* subsequent critical phonemes (e.g., /guː/-/t/ or /muː/-/t/). To adequately control for prompts including the critical phoneme, matching was based on both syllable frequency and syllable-*transition* frequency. For each parameter, Mann-Whitney *U* tests confirmed the absence of significant differences between stereotyped and unstereotyped prompts (always *z* ≤ 0.34, not significant). Moreover, stereotyped and unstereotyped prompts were congruent in terms of consonant-vowel structure, noun-verb classification, and meter (for details, see section “Linguistic materials”).

SD: Standard deviation

### Experimental conditions

Each patient was asked to produce prompts (e.g., /guː/) and target words (e.g., /tants/) with critical onsets (e.g., /t/) in six experimental conditions. These conditions were:

1. *no prompt* preceding the target word to estimate the actual severity of deficits in speech-motor planning (e.g., /tants/);
2. *schwa-syllable prompt* preceding the target word to explore the role of non-verbal phonation (e.g., /əː/-/tants/);
3. *stereotyped prompt* to determine the clinical potential of formulaic language resources (e.g., /guː/-/tants/, derived from the phrase “Guten Morgen” that, in its entirety, was not explicitly mentioned to the patient);
4. *unstereotyped prompt* serving as non-formulaic control utterance for the previous task (e.g., /muː/-/tants/, derived from the adjective “Mutig”);
5. *communicative-pragmatic prompt* to investigate the benefit from revealing the origin of the stereotyped prompt in a conversational context (e.g., the experimenter repeatedly used the phrase “Guten Morgen” to address the patient who, without repeating the formulaic expression as a whole, produced /guː/-/tants/ subsequently); and
6. *active-motor prompt* to measure the influence of articulatory priming (e.g., the patient repeatedly used the complete phrase “Guten Morgen” and then produced /guː/-/tants/; for an overview of the experimental conditions, see Table 3).

**Table 3:** Experimental conditions.

	1. No prompt	2. Schwa-syllable prompt	3. Stereotyped prompt	4. Unstereotyped prompt	5. Comm.-pragmatic prompt	6. Active-motor prompt
**Prompt**	—	/əː/	/guː/	/muː/	/guː/	/guː/
**Critical word onset**	/t/	/t/	/t/	/t/	/t/	/t/
**Prompt and target word**	/tants/	/əː/-/tants/	/guː/-/tants/	/muː/-/tants/	/guː/-/tants/	/guː/-/tants/
**Underlying phrase or word**	—	—	“Guten Morgen”	“Mutig”	“Guten Morgen”	“Guten Morgen”
**Embedding phrase in conversational context**	No	No	No	No	Yes	No
**Producing entire phrase before using it as prompt**	No	No	No	No	No	Yes

Overview of the experimental conditions. Testing materials included 10 non-formulaic one-syllable target words (nouns from the middle-frequency spectrum; e.g., “Tanz”), 16 formulaic expressions (e.g., “Guten Morgen”), and 16 non-formulaic control words (e.g., “Mutig”).

As *baselines*, each patient produced the full repertoire of 16 formulaic phrases and 16 non-formulaic control words to compare the articulatory quality of critical syllable onsets (e.g., /t/ in “Guten Morgen” and “Mutig”).

### Procedures

Testing was administered in different sessions, one for each experimental condition and one for each baseline. Sessions were separated by one week to minimize carryover effects. To avoid systematic practice or fatigue effects due to stimulus order, the sequence of experimental conditions and baselines was randomly alternated across participants. In each session, patients were seated in front of two loudspeakers at a distance of 75 cm and listened to a playback. The playback included a metronome (68 beats per minute) to control for articulatory tempo that may affect verbal output in neurological communication disorders [29]. Throughout all conditions and baselines, the experimenter presented verbal utterances by reading them aloud (e.g., /guː/-/tants/). Patients heard each utterance once and then repeated it five times at the pace of the metronome, with a sound signaling when to speak. To increase the reliability of the testing, five repetitions per utterance were preferred to single trials. Patients did not directly face the experimenter to rule out the possibility of lip-reading. Utterances were audiotaped using a head microphone (C520Vocal Condenser Microphone, AKG Acoustics, Vienna, Austria). Testing duration ranged from 10 to 30 minutes per patient and session. Overall, we recorded 580 utterances per patient, resulting in 11600 utterances across participants.

### Data analysis

Two independent phoneticians evaluated critical onsets within the recorded utterances (e.g., first /t/ in /guː/-/tants/). The scoring system was as follows: a maximum of two points for each correct critical onset; one point for each incorrect critical onset that was, however, correct with respect to manner and place of articulation (e.g., phoneme realization /d/ instead of /t/); no points for any further utterances or omissions (different phoneme realization: 31.9%; unintelligible phoneme realization: 22.6%; no phonation: 45.5%). This two-level scoring system resulted in high inter-rater reliability in the current study (*r* = 0.98) and proved to be robust in previous work [30]. We calculated percentages of correctly produced critical onsets for each experimental condition and baseline per patient. Percentages reflect averages of all scores obtained by the two phoneticians. These mean percentages were used in a random-intercept mixed-model analysis with two fixed factors: Experimental Condition (no prompt; schwa-syllable prompt; stereotyped prompt; unstereotyped prompt; communicative-pragmatic prompt; and active-motor prompt) and Stimulus Order (to account for potential practice or fatigue effects). For direct comparisons between the experimental conditions, we performed paired-sample *t*-tests. All evaluations were conducted with two-tailed alpha levels of 0.05; for multiple comparisons, we applied the Bonferroni-Holm correction.

## Results

**Fig 1:**
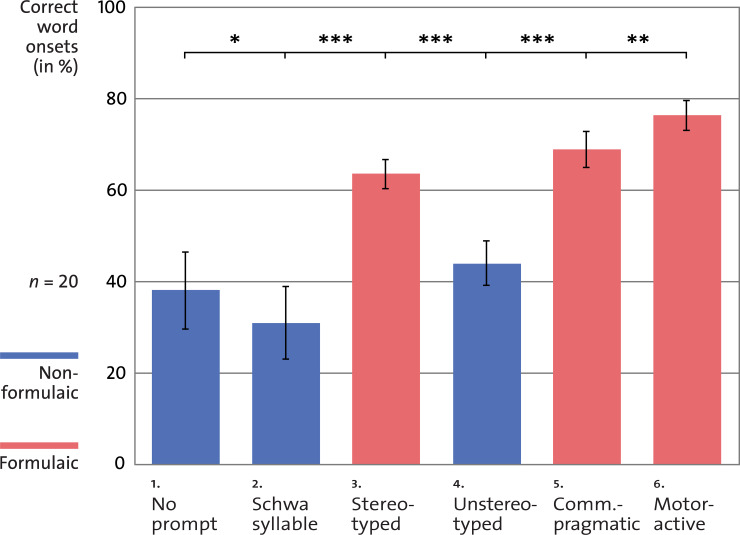
Means of correct word onsets (e.g., /t/ in “Tanz”) depending on previous verbal cues (from *left* to *right*): No prompts (e.g., /tants/), schwa-syllable prompts (e.g., /əː/-/tants/), stereotyped prompts (e.g., /guː/-/tants/, derived from formulaic phrase “Guten Morgen”), unstereotyped prompts (e.g., /muː/-/tants/, derived from non-formulaic control word “Mutig”), communicative-pragmatic prompts (e.g., /guː/-/tants/, produced immediately after the experimenter uses the underlying phrase “Guten Morgen” in a conversational context), and motor-active prompts (e.g., /guː/-/tants/, produced immediately after the patient repeatedly uses the underlying phrase “Guten Morgen” as articulatory priming). Verbal cues originate either from formulaic expressions (shown in *red*) or from non-formulaic control words (shown in *blue*; for an overview of all experimental conditions, see Table 3). Statistical analyses revealed significant gains in correct word onsets after verbal cues with distinct features of formulaicity (stereotyped and communicative-pragmatic prompts), as well as after articulatory priming (motor-active prompts; * p < 0.05; ** p < 0.01; *** p < 0.001). Error bars refer to 95%-confidence intervals.

The random-intercept mixed-model analysis revealed a significant effect of Experimental Condition [*F*(5, 90) = 5.28, *p* < 0.001] and no significant effect of Stimulus Order [*F*(5, 90) = 1.99, *p* = 0.1]. To differentiate these findings, *primary* evaluations focused on the proportion of correctly produced critical onsets depending on the formulaicity of the preceding verbal cues. As specified above, linguistic criteria of formulaicity were: stereotypy [condition 3 *versus* condition 4] and embeddedness in communicative-pragmatic context [condition 3 *versus* condition 5] [3]. Paired-sample *t*-tests point to significant increases in correct word onsets after stereotyped prompts [condition 3 *versus* condition 4; *t*(19) = 9.63, *p* < 0.001, Cohen’s *d*_*z*_ = 2.2] and after communicative-pragmatic prompts [condition 3 *versus* condition 5; *t*(19) = 2.82, *p* = 0.01, Cohen’s *d*_*z*_ = 0.6; for means and confidence intervals, see Fig 1 and Table 4].

**Table 4:** Results.

**A. Experimental conditions**						
	**1. No prompt**	**2. Schwa-syllable prompt**	**3. Stereotyped prompt**	**4. Unstereotyped prompt**	**5. Comm.-pragmatic prompt**	**6. Active-motor prompt**
**Correct word onsets, in percent (CI)**	38.5 (10.8)	31.5 (8.3)	62.9 (7.0)	43.9 (6.5)	68.8 (7.7)	76.2 (7.3)
**B. Baseline performances**						
		**Baseline: formulaic expressions**		**Baseline: non-formulaic control words**		
**Correct syllable onsets, in percent (CI)**		77.8 (8.4)		57.6 (8.3)		

Means of correctly produced critical onsets for each experimental condition (A) and baseline performance (B; for details, see section “Experimental conditions” and Table 3).

CI: Confidence interval

*Secondary* exploratory evaluations addressed the role of articulatory priming [condition 5 *versus* condition 6], suggesting significantly improved word onsets after active-motor prompts [*t*(19) = 3.57, *p* = 0.002, Cohen’s *d*_*z*_ = 0.8]. Taking a closer look at the function of non-verbal phonation [condition 1 *versus* condition 2], the analyses yielded a significantly lower rate of correctly produced word onsets after schwa-syllable prompts [*t*(19) = 2.15, *p* = 0.04, Cohen’s *d*_*z*_ = 0.5; for means and confidence intervals, see Fig 1 and Table 4].

As expected, articulatory quality of critical syllable onsets was significantly higher for formulaic expressions than for non-formulaic control words, thus confirming the adequacy of the source material (e.g., “Guten Morgen” or “Mutig”) for developing the experimental prompts [e.g., /guː/ or /muː/; baseline comparison: *t*(19) = 10.71, *p* < 0.001, Cohen’s *d*_*z*_ = 2.4; for means and confidence intervals, see Table 4].

## Discussion

The present proof-of-concept study aimed to investigate whether or not using preserved formulaic language skills can relieve word-onset difficulties in apraxia of speech. A total of 20 left-hemisphere stroke patients with chronic moderate-to-severe apraxia of speech were asked to produce German target words (e.g., “Tanz”) with critical onsets (e.g., /t/). Prior to each target word, the patients produced verbal cues that differed in standard features of formulaicity: (i) stereotypy [condition 3 *versus* condition 4] and (ii) embeddedness in communicative-pragmatic context [condition 3 *versus* condition 5] [3]. Moreover, cues varied in articulatory priming [condition 5 *versus* condition 6] and in non-verbal phonation [condition 1 *versus* condition 2]. Aside from these experimental alterations, cues were matched for consonant-vowel structure, syllable-transition frequency, noun-verb classification, meter, and articulatory tempo (for means of linguistic parameters, see Table 2; for an overview of all experimental conditions, see Table 3). Statistical analyses revealed significant gains in correctly produced word onsets after verbal cues with high stereotypy [*p* < 0.001, Cohen’s *d*_*z*_ = 2.2] and embeddedness in communicative-pragmatic context [*p* = 0.01, Cohen’s *d*_*z*_ = 0.6]. Over and above language formulaicity, these gains were even more pronounced after articulatory priming [*p* = 0.002, Cohen’s *d*_*z*_ = 0.8], whereas cues of non-verbal phonation affected the quality of critical word onsets in a negative way [*p* = 0.04, Cohen’s *d*_*z*_ = 0.5]. In summary, these results indicate that preserved formulaic language skills and articulatory priming can have an immediate positive impact on word-onset difficulties in apraxia of speech.

Our data point to an articulatory benefit from *stereotyped* prompts derived from formulaic expressions—that is, knowledge about the origin of verbal cues was *implicit* (e.g., the experimenter did not mention the underlying phrase “Guten Morgen” to the patient who produced /guː/-/tants/; see Table 3). Notably, the effect size resulting from stereotyped prompts appeared to be large (Cohen’s *d*_*z*_ = 2.2). It could be argued that this strong effect emerges from linguistic variables other than language formulaicity, such as syllable frequency (i.e., total occurrence of prompts in German; e.g., /guː/) or syllable-transition frequency (i.e., total occurrence of prompts *and* subsequent critical phonemes in German; e.g., /guː/-/t/). However, our design carefully controlled for syllable frequency, syllable-transition frequency, and similar parameters (for details, see Table 2). As an explanation for the superiority of stereotyped prompts (e.g., /guː/), we presume that this type of verbal cue triggered, in a holistic manner, the retrieval of speech-motor patterns necessary to complete the remaining syllables of formulaic expressions (e.g., /tn̩ ˈmɔʁɡn̩/). In our experiment, the anticipated speech-motor patterns (e.g., /ˈɡuːtn̩ ˈmɔʁɡn̩/) were only partially executed to facilitate critical word onsets (e.g., /t/) of divergent target utterances (e.g., /tants/). Consistent with the idea that stereotyped prompts rely on holistic properties of formulaic language, we noticed in our testing sessions that some patients occasionally had problems to stop the production of entire phrases once the articulatory process was started. Likewise, it may be worthwhile to consider the amount of articulatory errors within stereotyped prompts (e.g., incorrect /g/ in /ɡuː/) as opposed to unstereotyped prompts (e.g., incorrect /m/ in /muː/). For example, holistic processing of formulaic language may especially lower the probability of errors within stereotyped prompts, which in turn may diminish articulatory distraction and improve the quality of the following target utterances—a hypothesis to be consolidated in future research.

Our results yielded an increase in correct word onsets after *communicative-pragmatic* prompts—that is, knowledge about the origin of verbal cues was *explicit* (e.g., the experimenter used the phrase “Guten Morgen” to address the patient who, subsequently, produced /guː/-/tants/; see Table 3). This increase through communicative-pragmatic prompts corresponds to a medium effect size (Cohen’s *d*_*z*_ = 0.6). We propose that there may be an added value associated with engagement of the language network and, potentially, with relatedness to conversational context when identifying the origin of verbal cues. Support for the latter notion comes from data demonstrating an advantage of aphasia therapy protocols that require embedding target utterances in social interaction (e.g., requesting objects) compared to non-communicative exercises (e.g., confrontation naming) [31]. Moreover, anecdotal evidence implies that preserved language skills in aphasia are most apparent in communicative-pragmatic context (e.g., “My poor Jacqueline, I don’t even know your name!”) [32]. In analogy to aphasia research, our results emphasize the promising role of communicative-pragmatic context along with language stereotypy in clinical trials on apraxia of speech.

As another interesting exploratory finding of our study, articulatory priming proved to increase the benefit from verbal cueing (large positive effect: Cohen’s *d*_*z*_ = 0.8), while non-verbal phonation *per se* led to the lowest rate of correct syllable onsets across all experimental conditions (moderate negative effect: Cohen’s *d*_*z*_ = 0.5; see Fig 1 and Table 4). Hence, repeated production of formulaic expressions (e.g., “Guten Morgen”) prior to their use as motor-active prompts (e.g., /guː/-/tants/) may be a helpful add-on strategy to boost the articulatory outcome of cueing techniques. In contrast, the non-favorable influence of schwa-syllable prompts is congruent with the claim that the success of cueing techniques arises from higher-level language processing rather than simple voicing (e.g., phoneme realization /əː/). Accordingly, modern linguistic theories tend to interpret symptoms in apraxia of speech as deficits in higher-level language processing [1].

The current results indicate that language formulaicity may be a valuable resource in clinical practice. However, the cross-sectional design does not allow conclusions with regard to symptom recovery over time. A longitudinal section will be needed to substantiate the possible long-term effect of using preserved formulaic language skills in patients with apraxia of speech. This is particularly true when comparing the proposed keyword technique based on language formulaicity with established methods in speech-language therapy, among them audiovisual, tactile-kinesthetic or gestural cues [16] alongside more recent approaches [17]. To our knowledge, there is no evidence from randomized controlled trials on the outcome of treatment programs focusing on word-onset production. Subsequent research will be required to determine the most appropriate attempt to permanently alleviate word-onset difficulties.

Although limited by the cross-sectional design, our results still suggest that using preserved formulaic language skills may enable patients to overcome word-onset difficulties in apraxia of speech. More specifically, patients may be capable of retrieving intact speech-motor sequences within formulaic expressions in order to restore incorrect initial segments of non-formulaic verbal utterances. As mentioned previously, formulaic language is known for relying on right-hemisphere frontotemporal and bilateral subcortical neural networks [11–15]. If indeed preserved formulaic language skills are suitable to reduce failures in speech-motor planning, neuroscience data will have to clarify how such benefits relate to the interplay of left perilesional and right intact language networks in post-stroke rehabilitation. In this context, the present behavioral results may establish an empirical basis for future research on treatment-induced neuroplasticity of language function. Just as importantly, our results will hopefully inspire clinical trials on preserved formulaic language skills as a potential means to promote recovery from apraxia of speech.
